# ER stress abrogates the immunosuppressive effect of IL-10 on human macrophages through inhibition of STAT3 activation

**DOI:** 10.1007/s00011-019-01261-9

**Published:** 2019-06-21

**Authors:** Ivo S. Hansen, Josca M. Schoonejans, Lathees Sritharan, Johan A. van Burgsteden, Carmen A. Ambarus, Dominique L. P. Baeten, Jeroen den Dunnen

**Affiliations:** 1 Amsterdam Rheumatology and Immunology Center, Location Academic Medical Center, Meibergdreef 9, 1105 AZ Amsterdam, The Netherlands; 2 Department of Experimental Immunology, Amsterdam Infection and Immunity Institute, Amsterdam UMC, University of Amsterdam, Meibergdreef 9, 1105 AZ Amsterdam, The Netherlands

**Keywords:** Macrophages, ER stress, Inflammation, Cytokines, Signal transduction

## Abstract

**Objective and design:**

To determine whether ER stress affects the inhibitory pathways of the human immune system, particularly the immunosuppressive effect of IL-10 on macrophages.

**Material or subjects:**

In vitro stimulation of human monocyte-derived macrophages.

**Treatment:**

Cells were stimulated with TLR ligands and IL-10, while ER stress was induced using thapsigargin or tunicamycin.

**Methods:**

mRNA expression was determined using qPCR, while cytokine protein production was measured using ELISA. Protein expression of receptors and transcription factors was determined using flow cytometry. Student’s t test was used for statistics.

**Results:**

While under normal conditions IL-10 potently suppresses pro-inflammatory cytokine production by LPS-stimulated macrophages, we demonstrate that ER stress counteracts the immunosuppressive effects of IL-10, leading to increased pro-inflammatory cytokine production. We identified that ER stress directly interferes with IL-10R signaling by reducing STAT3 phosphorylation on Tyr705, which thereby inhibits the expression of SOCS3. Moreover, we show that ER stress also inhibits STAT3 activation induced by other receptors such as IL-6R.

**Conclusions:**

Combined, these data uncover a new general mechanism by which ER stress promotes inflammation. Considering its potential involvement in the pathogenesis of diseases such as Crohn’s disease and spondyloarthritis, targeting of this mechanism may provide new opportunities to counteract inflammation.

## Introduction

The mechanisms triggering immune activation and inflammation during normal host defense responses are currently well understood. This includes the rapid recognition of pathogens by the innate immune system, which is mediated by detection of molecular patterns expressed as structural component of microorganisms by various innate immune cells. These molecular patterns, such as lipopolysaccharide (LPS) or flagellin, are recognized by a group of receptors called pattern recognition receptors (PRR) [1]. Ligation of molecular patterns to their respective receptors on innate immune cells leads to activation of receptor-specific signaling cascades inducing mostly pro-inflammatory genes tailored towards the type of pathogen that is encountered.

Significant progress has also been made in our understanding of how this inflammatory cascade, once initiated, is regulated and ultimately resolved in order to avoid that the protective response towards the invading microorganisms results in uncontrolled and/or chronic tissue inflammation. Crucial regulators of innate immune responses are anti-inflammatory cytokines such as IL-10. Mice lacking IL-10 spontaneously develop severe enterocolitis [2, 3]. Likewise, infants with loss-of-function mutations in the IL-10R are unable to downregulate LPS-induced macrophage activation and suffer from dramatic inflammatory bowel disease [4].

The latter observation suggests that lack of appropriate regulation and resolution in inflammatory responses may not only be relevant in the context of host defense but also in chronic inflammatory disorders. This mechanism may be more relevant to so-called ‘hyper-inflammatory’ diseases, which are driven by tissue-specific abnormal innate immune responses to various types of cellular stress, rather than by classical acquired immune responses to autoantigens [5]. These hyper-inflammatory diseases include rare monogenic disorders, such as the fever syndromes due to exaggerated IL-1 production [6], as well as polygenic diseases such as Crohn’s disease and spondyloarthritis [7].

A particular form of stress that has been recently recognized to play a role in a variety of chronic inflammatory conditions is endoplasmic reticulum (ER) stress [8]. The ER plays an essential role in the highly regulated process of protein production. High protein production and/or disturbances in protein assembly can lead to an accumulation of unfolded or misfolded proteins in the ER, which induces ER stress and subsequently the so-called unfolded protein response. This is a highly conserved pathway mediated by the kinases IRE1 and PERK that causes an immediate reduction in protein synthesis as well as an increase in protein folding capacity. Whereas this physiological process happens in all cell types, it has become clear in the last years that ER stress plays an important role in shaping of immune responses. For example, immune cells have been shown to be dependent on ER stress proteins during cell differentiation [9–12]. In addition, ER stress has a direct effect on pro-inflammatory cytokine production by macrophages, since induction of ER stress has been shown to strongly potentiate LPS-induced pro-inflammatory cytokine transcription [13, 14]. Furthermore, in macrophages TLR ligation activates the XBP1 pathway that is necessary for a proper immunological response [15].

In chronic inflammatory conditions, ER stress has mainly been linked to Crohn’s disease and ulcerative colitis, the two main forms of inflammatory bowel disease (IBD). Of the various polymorphisms that are associated with (IBD), several are associated with ER stress components. *XBP1* knock-outs show a greater susceptibility to enterocolitis in mice, which was confirmed in human IBD-patients [16, 17]. ER stress has also been linked to spondyloarthritis, where misfolding of HLA-B27 in the ER is hypothesized to potentiate the production of IL-23 [18]. Whereas these data implicate a role for ER stress in triggering inflammation in these conditions, alternatively ER stress may also affect the regulation and resolution of inflammation. Therefore, in this study we set out to investigate the effects of ER stress on the regulatory effects of the prototypical anti-inflammatory cytokine, i.e., IL-10, on the inflammatory response of myeloid cells.

## Materials and methods

### Ethics statement

This study was done according to the ethical guidelines of the Academic Medical Center and human material was obtained in accordance with the AMC Medical Ethics Review Committee according to the Medical Research Involving Human Subjects Act. Buffy coats obtained after blood donation (Sanquin) are not subjected to informed consent, which is according to the Medical Research Involving Human Subjects Act and the AMC Medical Ethics Review Committee. All samples were handled anonymously.

### Cells

In vitro differentiated macrophages were obtained by isolation of monocytes from buffy coats (Sanquin Blood Supply) by density gradient centrifugation using Lymphoprep (Nycomed) and Percoll (Pharmacia). Macrophages were differentiated by culturing the monocytes for 6 days in IMDM (Lonza) containing 5% FBS (Biowest) and 86 µg/mL gentamicin (Gibco), supplemented with 20 ng/mL GM-CSF (Invitrogen). For dendritic cells, medium was additionally supplemented with 2 ng/mL IL-4 (Miltenyi Biotec). At day 2 or 3 half of the medium was replaced with new medium containing cytokines.

### Stimulation

Macrophages were harvested by removing medium and washing the cells with PBS and adding TrypLE select (Invitrogen). Macrophages were pre-treated with 10 µM thapsigargin (Calbiochem) or 10 µg/mL tunicamycin (Sigma Aldrich) for 2 h at 37 °C to induce ER stress. Cells (30,000–50,000 per well) were stimulated in 96-well plates (Corning) with 100 ng/mL LPS (from *E. coli* o111:B4; Sigma Aldrich), 20 µg/mL Poly I:C (Sigma Aldrich), 10 µg/mL Pam3CSK4 (Invivogen), 10 µg/mL MDP (Invivogen), 10 µg/mL curdlan (from *Alcaligenes faecalis*; Sigma Aldrich), 5 ng/mL IL-10 (Miltenyi Biotec), and 25 ng/mL IL-6 (R&D systems).

For analysis of the cytokines TNF, IL-6, and IL-23, cells were stimulated for 24 h and supernatant was stored at − 20 °C until measured by ELISA. Cytokine levels were measured using antibody pairs for TNF (eBioscience), IL-6, and IL-23 (both UcyTech).

### Quantitative real-time PCR

To analyze mRNA levels, cells were lysed after indicated time of stimulation and subsequently mRNA extraction was performed using RNeasy Mini Kit (Qiagen) and cDNA synthesis using RevertAid H Minus First Strand cDNA Synthesis Kit (Fermentas). Quantitative real-time PCR (StepOnePlus Real-Time PCR System; Thermo Fisher Scientific) was performed using Taqman Master Mix and Taqman primers (both from Thermo Fisher Scientific). Primers used were: *GAPDH* (4310884E), *IL12A* (Hs01073447_m1), *IL12B* (Hs01011518_m1), *IL23A* (Hs00372324_m1), *IL6* (Hs00174131_m1), *SOCS3* (Hs02330328_s1), and *TNF* (Hs00174128_m1).

### Flow cytometry

Macrophages were stained after inducing ER stress for IL-10R expression using anti-IL10Rα-PE antibody (CD210, REA239, Miltenyi Biotec) in PBS containing 0.5% BSA. For intracellular staining cells were stimulated with IL-10 or IL-6 for 15 min and subsequently, fixed with 4% paraformaldehyde (Thermo Scientific) for 15 min at 37 °C washed and permeabilized using ice-cold methanol for at least 60 min at − 20 °C [19]. Staining was done using the following antibodies: anti-pSTAT3(S727)-PE (558557; BD Biosciences), anti-pSTAT3(Y705) (9145S; Cell Signaling), anti-STAT3 (12640S; Cell Signaling), anti-pSTAT3(S727) (94994S; Cell Signaling), and anti-STAT3-FITC (IC1799F; R&D Systems). Fluorescence was measured using a FACSCanto II (BD Biosciences).

### Data analysis

Data were analyzed for statistical significance using paired Student’s *t* test with GraphPad Prism version 5.01 software (GraphPad Software).

## Results

### ER stress counteracts the immunosuppressive effect of IL-10 on human LPS-stimulated macrophages

**Fig. 1:**
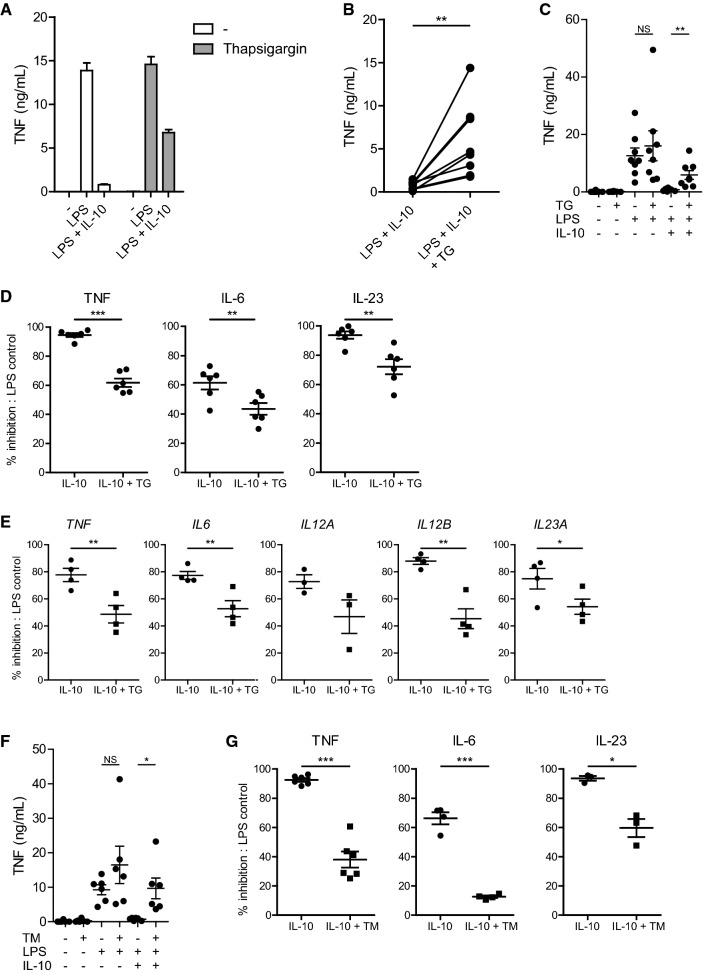
ER stress counteracts the immunosuppressive effect of IL-10 on human LPS-stimulated macrophages. **a** Representative example of TNF production by macrophages that were stimulated with LPS in combination or without IL-10 after pre-treatment with thapsigargin (TG) or vehicle control. Mean + SEM of triplicates. **b** TNF production by macrophages stimulated with LPS in combination with IL-10 after pre-treatment with TG or vehicle control. Each pair of dots represents one donor. **c** TNF production by macrophages stimulated with LPS in combination with IL-10 after pre-treatment with TG or vehicle control. Each dot represents mean of one donor, mean + SEM. **d** Macrophages were stimulated with LPS in combination with IL-10 after pre-treatment with TG or vehicle control. Data represented here is the percentage of inhibition of pro-inflammatory cytokine production by IL-10 of LPS-stimulated cells: (1 − (LPS + IL-10)/(LPS)) × 100%. Each dot represents one donor, mean + SEM. **e** Macrophages were stimulated with LPS in combination with IL-10 after pre-treatment with TG or vehicle control. Cells were lysed after 6 h and mRNA expression was measured using qPCR for indicated genes. Data represented here is the percentage inhibition of LPS-stimulated cells. Each dot represents one donor, mean + SEM. **f** TNF production by macrophages stimulated with LPS in combination with IL-10 after pre-treatment with tunicamycin (TM) or vehicle control. Each dot represents mean of one donor, mean + SEM. **g** Macrophages were stimulated with LPS in combination with IL-10 after pre-treatment with tunicamycin (TM) or vehicle control. Data represented here are the percentage inhibition of LPS-stimulated cells, similar as 1D. Each dot represents one donor, mean + SEM. Experiments (**a**–**d**, **f**, **g**) were performed in triplicate. After 24 h supernatants were analyzed using ELISA. *p < 0.05, **p < 0.01, ***p < 0.001, *NS* not significant, Student’s *t* test

To assess if ER stress impacts the regulation of inflammation, we investigated the effect of IL-10 on inflammatory macrophage responses in the presence or absence of ER stress. In line with literature, stimulation of human GM-CSF differentiated macrophages with LPS induced the production of pro-inflammatory cytokine TNF, which was potently inhibited by the addition of IL-10 (Fig. 1a). However, induction of ER stress by pre-treatment with thapsigargin impaired the suppressive effect of IL-10 on TNF production (Fig. 1a). When selectively comparing the condition of LPS + IL-10 stimulation for multiple donors, ER stress led to a strong increase in the net production of TNF by human macrophages (Fig. 1b). As a control, we verified that ER stress did not affect TNF production induced by LPS stimulation in absence of IL-10 (Fig. 1c). Quantification of the inhibition revealed a significant decrease in the capacity of IL-10 to inhibit LPS-induced production of TNF, but also other pro-inflammatory cytokines such as IL-6 and IL-23 (Fig. 1d).

To confirm and extend these findings, we first assessed whether the same effects were seen at the level of gene transcription. Analysis of mRNA expression of LPS-stimulated cells after 6 h using quantitative real-time PCR confirmed that key pro-inflammatory cytokine transcripts were strongly reduced after addition of IL-10. However, this inhibition of pro-inflammatory cytokine transcription by IL-10 was again impaired by ER stress, to a similar degree as seen on protein level (Fig. 1e).

To further confirm that the observed effects truly result from induction of ER stress, we assessed the effect of tunicamycin, another potent inducer of ER stress. Tunicamycin reduced the suppressive effect of IL-10 in a similar manner as thapsigargin, as shown by protein production of TNF (Fig. 1f) and quantification of inhibition for TNF, IL-6, and IL-23 (Fig. 1g).

Taken together, these data demonstrate that ER stress counteracts the immunosuppressive effect of IL-10 on inflammatory cytokine production by LPS-stimulated macrophages.

### ER stress inhibits IL-10-induced STAT3 Tyr705 phosphorylation

**Fig. 2:**
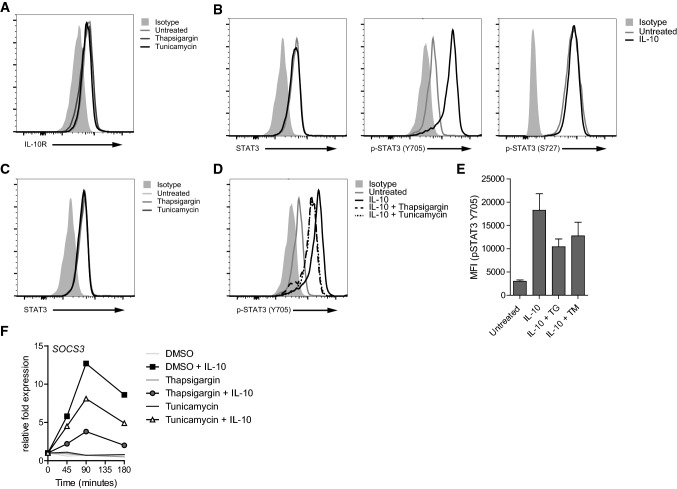
ER stress inhibits IL-10-induced STAT3 Tyr705 phosphorylation. **a** Macrophages were analyzed for IL-10 receptor (IL10R) expression after treatment with thapsigargin (TG), tunicamycin (TM), or vehicle control for 2 h using flow cytometry. Light grey histogram indicates background staining. **b** Cells were measured for expression of STAT3 and phosphorylation of STAT3 at Tyr705 and Ser727 after stimulation with 5 ng/mL IL-10 for 15 min using flow cytometry. Light grey histogram indicates background staining. **c** Macrophages were treated with TG, TM, or vehicle control for 2 h and analyzed for STAT3 expression. Light grey histogram indicates background staining. **d**, **e** Macrophages were pre-treated with TG, TM, or vehicle control and stimulated with 5 ng/mL IL-10 for 15 min. Phosphorylation of STAT3 at Tyr705 was analyzed using flow cytometry. Light grey histogram indicates background staining. Representative example (**d**) and pooled MFI data (**e**) from three different experiments, mean + SEM. **f** Macrophages were pre-treated with TG, TM or vehicle control and stimulated with 5 ng/mL IL-10 and analyzed for mRNA expression of *SOCS3* using qPCR (normalized to *GAPDH* expression, fold increase compared to unstimulated control). Data (**a**–**d**, **f**) are representative examples of three independent experiments using different donors

Next, we set out to investigate the mechanism of reduced IL-10-mediated effects during ER stress. First, we examined if ER stress affects the expression of the IL-10 receptor (IL-10R) on human macrophages. Neither thapsigargin nor tunicamycin decreased IL-10R surface expression as assessed by flow cytometry (Fig. 2a).

A key event of IL-10R signaling is phosphorylation and subsequent dimerization of STAT3 [20, 21]. We analyzed STAT3 phosphorylation using flow cytometry, which enabled us to quantify phosphorylation differences, but also to determine whether STAT3 phosphorylation occurs in all cells or only in particular subpopulations. To specifically study the impact of ER stress on IL-10-mediated signaling we stimulated the cells with IL-10 alone, i.e., in the absence of LPS. In agreement with the literature [22], stimulation of macrophages with IL-10 led to the universal phosphorylation of STAT3 on tyrosine 705 (Y705), while it did not induce phosphorylation of STAT3 on serine 727 (S727) (Fig. 2b). Whereas neither thapsigargin nor tunicamycin had any effect on the expression of total STAT3 (Fig. 2c), both ER stress inducers reduced IL-10-induced STAT3 Y705 phosphorylation (Fig. 2d, e). Since SOCS3 is one of the main regulators of IL-10R signaling [23–25], we next set out to investigate the induction of SOCS3. Indeed, thapsigargin and tunicamycin strongly suppressed IL-10-induced mRNA expression of *SOCS3* (Fig. 2f), one of the main downstream effector molecules of IL-10R signaling that is dependent on STAT3-induced gene transcription [23, 24].

Combined, these data indicate that ER stress interferes with IL-10 signaling by suppressing phosphorylation of STAT3 on Y705, leading to strongly reduced levels of SOCS3.

### ER stress-induced suppression of STAT3 phosphorylation is not restricted to IL-10 signaling

**Fig. 3:**
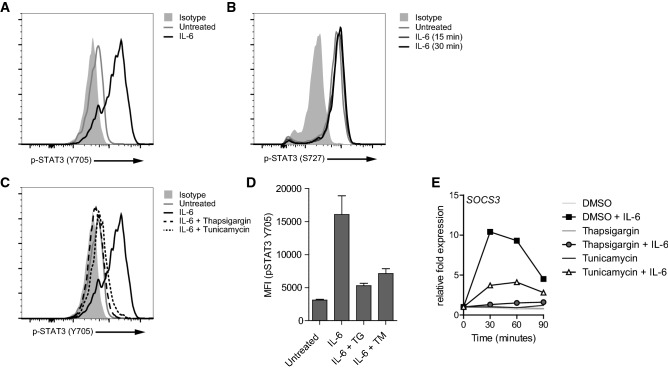
ER stress-induced suppression of STAT3 phosphorylation is not restricted to IL-10 signaling. Macrophages were stimulated with 25 ng/mL IL-6 for 15 min (**a**, **b**) or 30 min (**b**) and analyzed for phosphorylation of STAT3 at Tyr705 (**a**) and Ser727 (**b**) using flow cytometry. Light grey histogram indicates background staining. **c**, **d** Macrophages were pre-treated with thapsigargin (TG), tunicamycin (TM), or vehicle control and stimulated with 25 ng/mL IL-6 for 15 min. Phosphorylation of STAT3 at Tyr705 was analyzed using flow cytometry. Light grey histogram indicates background staining. Representative example (**c**) and pooled MFI data (D) from three different experiments, mean + SEM. **e** Macrophages were pre-treated with TG, TM or vehicle control and stimulated with 25 ng/mL IL-6 and analyzed for mRNA expression of *SOCS3* using qPCR (normalized to *GAPDH* expression, fold increase compared to unstimulated control). Data (**a**–**d**) are representative examples of three independent experiments using different donors

STAT3 is not only involved in IL-10 signaling, but also in signaling of receptors for several other cytokines, such as IL-6, IL-22 and OSM [26]. To determine whether ER stress specifically suppresses STAT3 activation induced by IL-10R signaling, or whether it also suppresses STAT3 activation induced by other cytokine receptors, we assessed the effect of thapsigargin and tunicamycin on STAT3 phosphorylation after stimulation with IL-6. Similar to IL-10, stimulation of macrophages with IL-6 induced STAT3 phosphorylation at Y705 (Fig. 3a), with only discrete impact on phosphorylation of STAT3 on S727 (Fig. 3b). Importantly, induction of ER stress by both thapsigargin and tunicamycin strongly reduced IL-6-induced phosphorylation of STAT3 at Y705 (Fig. 3c, d). Downstream of STAT3, IL-6-mediated *SOCS3* gene transcription was also suppressed after treatment with thapsigargin or tunicamycin (Fig. 3e). These data demonstrate that the inhibition of STAT3 activation by ER stress is not specific for IL-10R, but also suppresses STAT3 activation induced by other cytokine receptors.

### ER stress-induced suppression of IL-10 immunoregulation is not restricted to TLR4 or to macrophages

**Fig. 4:**
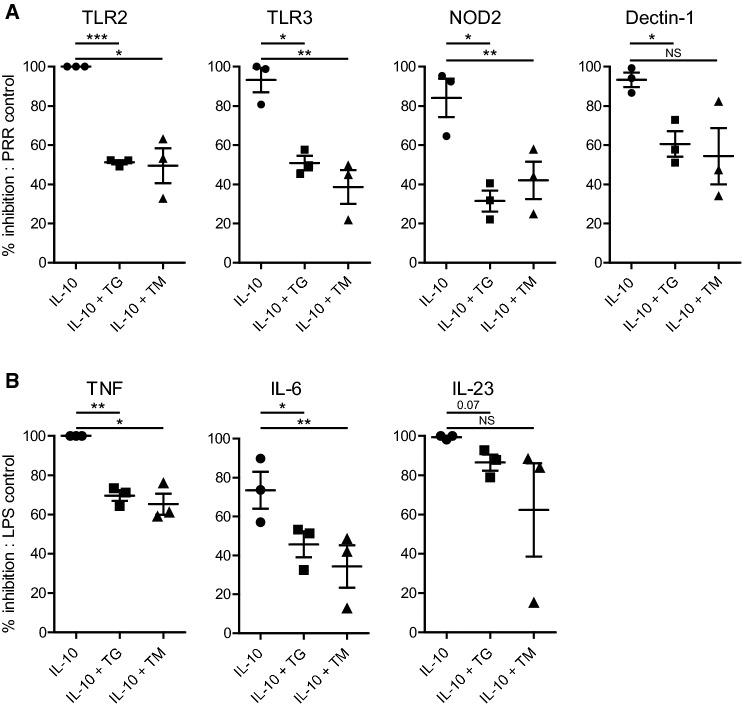
ER stress-induced suppression of IL-10 immunoregulation is not restricted to TLR4 or to macrophages. **a** Macrophages were stimulated with different PRR ligands with or without IL-10, after pre-treatment with thapsigargin (TG), tunicamycin (TM), or vehicle control. Stimulated receptors were TLR2 (Pam3CSK4), TLR3 (Poly I:C), NOD2 (MDP), and Dectin-1 (Curdlan). Data represented here is the percentage of inhibition of TNF production by IL-10 of LPS-stimulated cells: (1 − (LPS + IL-10)/(LPS)) x 100%. Each dot represents one donor, mean + SEM. **b** Dendritic cells were stimulated with LPS in combination or without IL-10 after pre-treatment with TG, TM, or vehicle control. Data represented here is the percentage inhibition of pro-inflammatory cytokine production by LPS-stimulated cells (similar to **a**). Each dot represents one donor, mean + SEM. Experiments were performed in triplicate. After 24 h supernatants were analyzed using ELISA. *p < 0.05, **p < 0.01, ***p < 0.001, *NS* not significant, Student’s *t* test

To determine whether ER stress only counteracts the immunosuppressive effect of IL-10 induced by TLR4 signaling, or whether it also impairs IL-10-mediated effects induced by other PRRs, we assessed the effect of several different PRR ligands. As shown in Fig. 4a, ER stress counteracted IL-10-induced suppression of TNF production after stimulation of various different PRRs, including TLR2 (using Pam3CSK4), TLR3 [using Poly (I:C)], NOD2 (using MDP), and Dectin-1 (using curdlan). Next, we examined whether the effect of ER stress was specific for macrophages, or whether it was also functional in other human immune cells. As shown in Fig. 4b, ER stress also reduced the suppressive effect of IL-10 on human dendritic cells (DC). These data demonstrate that ER stress counteracts the immunosuppressive effect of IL-10 upon stimulation of various different families of PRRs and in different cell types.

## Discussion

**Fig. 5:**
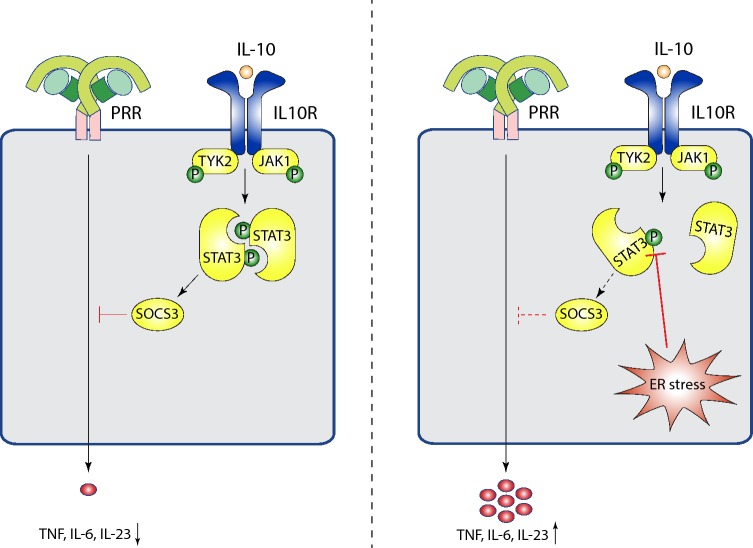
Model of enhanced pro-inflammatory cytokine production by ER stress-induced inhibition of IL-10 signaling. In the absence of ER stress, IL-10 potently inhibits PRR-induced cytokine production through recognition by the IL-10 receptor and subsequent STAT3 Tyr705 phosphorylation leading to SOCS3 production. In contrast, when cells are undergoing ER stress, STAT3 Tyr705 phosphorylation is inhibited, resulting in abrogation of the immunosuppressive effect of IL-10 and increased pro-inflammatory cytokine production

Regulation of inflammatory responses via key anti-inflammatory pathways such as IL-10 is essential to prevent uncontrolled and/or chronic tissue inflammation. Importantly, here we show that macrophages that are undergoing ER stress are less susceptible to the immunosuppressive effects of IL-10. Consequently, this reduced inhibitory effect of IL-10 leads to strongly increased production of pro-inflammatory cytokines such as TNF, IL-6 and IL-23. Furthermore, we identified that ER stress directly interferes with IL-10R signaling by reducing the phosphorylation of STAT3 on Tyr705, which thereby prevents expression of SOCS3 (see Fig. 5 for model).

IL-10 is one of the main cytokines responsible for inducing anti-inflammatory responses, which affects both innate and adaptive immune cells by limiting proliferation, cytokine production and costimulatory molecule expression [27, 28]. Mice deficient in either IL-10 or IL-10R spontaneous develop colitis [2], but only after colonization with microorganisms [3], showing the essential role that IL-10 plays after activation of immune cells by PRRs. Furthermore, IL-10R deficiency that was restricted to the macrophage population in the intestine was shown to induce spontaneous colitis [29]. In this study, we show that ER stress makes immune cells less susceptible to IL-10-induced inhibition of different families of PRRs. Since ER stress has been implicated in several chronic inflammatory disorders, the inability of immune cells to be inhibited during inflammation could play an important role in the pathogeneses of these diseases.

ER stress is strongly associated with pro-inflammatory cytokine production in various immune cells, but particularly in macrophages [30]. Components of the ER stress response such as XBP1 are known to be directly involved in signaling by TLRs, and lack of XBP1 results in impaired TNF, IL-6 and interferon β cytokine production [15]. In addition, ER stress has been linked to production of CXCL1/CXCL2 and IL-1β production through interaction with RIPK1 and GSK-3β, respectively [13, 14]. Recently also Notch has been described to be involved in ER stress [31], indicating that ER stress activates multiple pathways to enhance PRR activation. In addition to directly amplifying PRR-induced responses, we here identified the suppression of the inhibitory pathways of the immune system as a new mechanism by which ER stress promotes inflammation.

STAT3 controls immune regulation in a variety of immune cells. In neutrophils, knocking out STAT3 results in hyper-responsivity to stimulation [32]. Regulatory T cell function is also critically dependent on STAT3, since ablation of STAT3 in regulatory T cells leads to fatal intestinal inflammation mediated by Th17 cells [33]. In addition, STAT3 is a negative regulator of pro-inflammatory DC function, since specific knock-out of STAT3 in the DC population leads to general inflammatory conditions, most notably in the intestine of the animals [34]. Since in all these cell types ER stress is likely to occur during (chronic) immune activation, our finding that ER stress-induced STAT3 inhibition promotes inflammation may be applicable to a large variety of different immune cells. Interestingly, ER stress has previously been identified to suppress leptin-induced STAT3 activation in HEK293 cells, suggesting that this effect is not restricted to immune cells [35].

STAT3 is not only involved in IL-10R signaling, but also in signaling of receptors for several other cytokines. Intriguingly, our data show that ER stress does not only inhibit STAT3 phosphorylation after stimulation with IL-10, but also after stimulation with IL-6. These data indicate that ER stress-induced STAT3 inhibition may have broad implications, by affecting the signaling induced by multiple cytokines, including IL-10, IL-6, IL-22 and OSM [26, 36, 37]. Interestingly, while all the receptors of these cytokines signal via STAT3, the immunological effects of these cytokines are rather diverse, ranging from anti-inflammatory and wound-healing effects of IL-10 and IL-22 [38, 39] to more pro-inflammatory responses by OSM [40]. Therefore, the effect of ER stress-induced STAT3 inhibition will most likely be context-specific, depending on cytokine, cell type, and tissue involved. Interestingly, ER stress has previously also been shown to suppress the phosphorylation of STAT1 [41], suggesting that that inhibition of tyrosine phosphorylation by ER stress may be general feature for all STAT family members.

In this study, we have used SOCS3 as a read-out of STAT3 activation, but SOCS3 is also known to bind gp130 (which is part of the IL-6R complex) and prevent subsequent activation of STAT3, which functions as a negative feedback loop [42]. As such, SOCS3 could potentially play a role in ER stress-induced inhibition of STAT3 phosphorylation. However, since SOCS3 does not regulate IL-10R signaling [42–44], and we show that ER stress inhibits both IL-6 and IL-10 signaling, SOCS3 is less likely to play a role in ER stress-induced STAT3 inhibition. Alternatively, numerous other factors have been described to regulate STAT3 activity. For example, protein tyrosine phosphatase 1B (PTP1B), which is upregulated under ER stress conditions [45], has been described as a negative regulator of IL-10-induced STAT3 phosphorylation [46]. In addition, ER stress inhibits the activation of focal adhesion kinase (FAK), leading to reduced STAT3 phosphorylation at S727 and Y705 [47, 48]. Recently, it has also been identified that STAT3 can be phosphorylated at S754, which suppresses STAT3 activity [49]. Other options include TC45, a protein tyrosine phosphatase that regulates STAT3 by de-phosphorylation [50]. However, TC45 seems to require phosphorylation at S727 to mediate inhibition of STAT3 [51]. Interestingly, we only observed phosphorylation of STAT3 at S727 upon stimulation with IL-6, but not upon stimulation with IL-10. Therefore, TC45 activity may provide an explanation for the stronger ER stress-induced inhibition of STAT3 phosphorylation at Y705 that we observed upon stimulation with IL-6 compared to IL-10 (compare Fig. 2d and Fig. 3c). For future research it may also be valuable to assess the activation of the proximal kinases of STAT3, i.e., JAK1 and TYK2 [26], since impaired functioning of these kinases will also lead to suppressed phosphorylation of STAT3.

Albeit beyond the scope of the present study, the mechanisms described here could contribute to chronicity and/or exacerbation of tissue inflammation in a number of immune-mediated inflammatory diseases. First, there is clear genetic and functional evidence for a role of ER stress in IBD, such as the findings that loss of XBP1 is known to result in colonic inflammation [16, 17]. Interestingly, several findings suggest that the mechanism of ER stress-induced inflammation that we identified here, i.e., the suppression of inhibitory pathways, is also involved in chronic intestinal inflammation. Our data that ER stress reduces the sensitivity to IL-10 nicely corresponds with previous findings that show that lack of IL-10 induces colitis [2, 29]. Additionally, the reduced IL-10 sensitivity induced by ER stress particularly amplified the production of TNF and IL-23, two pro-inflammatory cytokines that have strong genetic associations with Crohn’s disease [52] and are effective therapeutic targets in IBD [53, 54]. Second, our findings could be relevant in the context of spondyloarthritis. Spondyloarthritis is strongly linked to expression of HLA-B27 [55], which is prone to misfolding during processing, leading to ER stress. Spondyloarthritis shares several genetic risk factors with Crohn’s disease, including TNF family and IL23R genes [55], and patients have an increased risk of developing colitis. While transgenic rats expressing human HLA-B27 show increased pro-inflammatory cytokine production by macrophages [18, 56], reduction of ER stress by reducing HLA-B27 misfolding through additional trans-gene expression of beta2-microglobulin in rats prevents intestinal inflammation in these animals. Interestingly, these rats still develop axial and peripheral arthritis [57], which highlights the specific importance of ER stress in controlling immune regulation in the intestine.

Taken together, we identified that ER stress abrogates the immunosuppressive effect of IL-10, which is mediated by inhibition of STAT3 phosphorylation. These data uncover a new mechanism by which ER stress promotes inflammation by multiple cell types and cytokines. Considering its potential involvement in the pathogenesis of diseases such as Crohn’s disease and spondyloarthritis, targeting of this mechanism may provide new opportunities to counteract inflammation in these diseases.
